# Supplementary material for: Local transcriptional covariation produces accurate estimates of cell phenotype
Source: Bioinformatics. 2026 Mar 31;42(5):btag135. doi: 10.1093/bioinformatics/btag135 (PMC13148962; doi:10.1093/bioinformatics/btag135)
Supplement: btag135_Supplementary_Data [file btag135_supplementary_data.zip › Sceodesic_Supplement_March_4th_2026_Resubmission_Formatting.pdf]

Runtimes and memory requirement

On a dataset of 100,000 cells, Sceodesic takes approximately 20 minutes on an Intel Xeon 3GHz CPU, with runtimes increasing roughly linearly with number of cells. If interpretability of the gene programs is less crucial, these runtimes can be improved up to 3.5x by replacing sparse PCA with regular PCA during PVD. Memory requirements scaled linearly with the size of the dataset, with only 25 GB required to construct program embeddings on a dataset of approximately 1 million cells.

Additional discussion of the $\mathcal{S}_+^n$ Riemannian manifold.

Symmetric positive definite matrices $\mathcal{S}_+^n$ of fixed dimension n can be viewed as a smooth convex submanifold of the space $\mathbf{R}^{n(n+1)/2}$ with the inherited euclidean metric. But the direct application of this metric on $\mathcal{S}_+^n$ is problematic, especially because it induces finite distance between points in $\mathcal{S}_n^+$ and matrices with zero or negative eigenvalues (Lin, 2019). Similarly, the Frechet mean obtained using the euclidean norm induces a swelling effect, where the determinant of the euclidean norm is strictly larger than the determinants of sample points (Arsigny et al., 2007). This might introduce unwanted distortions in our calculation of distance between the correlation matrices.

Many alternative norms have been developed to mitigate the finite-distance and swelling effects inherent in the Euclidean norms (Lin, 2019; Arsigny et al., 2007). The simplest substitute is to develop a norm in $\mathcal{S}_+^n$ that induces isometry in the smooth map $f : GL(n, \mathbb{R}) \rightarrow \mathcal{S}_+^n, f(A) = A^T A$ (Bhatia, 2009). The lower-bound approximation of the geodesic distance induced by this isometric norm is the logarithmic distance, given by $d_{\log}(A, B) = \| \log(A) - \log(B)\|_F$, where $A, B \in \mathcal{S}_+^n$ and $\log(A)$ represents the matrix logarithm of A. This $d_{\log}$ metric solves the finite-distance issue, as the distance between any point in $\mathcal{S}_+^n$ and matrices with zero or negative eigenvalues is infinity.

Method Details

Selection of Locally Variable Genes:

To ensure that the selection of highly variable genes is not dominated by the cells constituting larger cell cohorts, we utilize the notion of locally variable genes which allows for the selection of h distinct genes that are highly variable across and within cohorts, rather than only across the entire study. Consider n distinct cohorts c_1, c_2,..., c_n and a parameter h for the desired number of genes to select. Then, within each *cohort* i, we use Seurat's HVG selection procedure (implemented in Scanpy) to obtain the top h genes, which we denote by the set:

$$G_i = \{g_1^i, g_2^i, ..., g_h^i\}$$

We then score each gene by the number of times it appears in a cohort-specific HVG list, i.e. for a gene g:

$$s(g) = \sum_{i=1}^{n} \mathbb{I}_{g \in G_i}$$

We determine the final list of locally variable genes by selecting the h highest scoring genes.

Simulated Experiment Regulatory Network Structure

Below (Fig. 6) we provide the structure of the regulatory network structure used for the SERGIO simulations. Each cell sub-type was characterized by over-expression of a single master regulator (MR) and its downstream genes. Each cell type was characterized by two distinct cell-subtypes. Edge weights denote strength and direction of regulatory relationships.

Proof of the optimality of S_i^{log}

We now show that the solution $\hat{S}_i$ corresponding to

$$[\hat{S}_i^{log}]_{jj} = [\hat{U}^T \log(\tilde{\Sigma}_i)\hat{U}]_{jj} \quad \text{for } j = 1 \dots h$$

is the optimal feasible solution to the covariance matrix reconstruction problem:

$$\min_{S_i} \| \log(\tilde{\Sigma}_i) - \log(\hat{U} S_i \hat{U}^T)\|_F \quad \text{where } \tilde{\Sigma}_i = U_i \tilde{D}_i U_i^T,$$

subject to the constraint that S_i is diagonal. The objective function of interest, which minimizes the distance from the reconstruction matrix $\hat{U} S_i \hat{U}^T$ to the condition-normalized sample covariance matrix $\tilde{\Sigma}_i$ under the log-Euclidean metric, is equivalent to:

$$\min_{S_i} \| \log(\tilde{\Sigma}_i) - \hat{U} \log(S_i) \hat{U}^T \|_F$$

Factoring on the left and right yields:

$$\min_{S_i} \|\hat{U}(\hat{U}^T \log(\tilde{\Sigma}_i)\hat{U} - \log(S_i))\hat{U}^T)\|_F$$

But since the columns of $\hat{U}$ are orthonormal and do not change the norm of any matrix, the preceding minimization is equivalent to minimizing the simpler objective:

$$\min_{S_i} \|\hat{U}^T \log(\tilde{\Sigma}_i)\hat{U} - \log(S_i)\|_F$$

Setting $\log(S_i) = S_i^{log}$, we have:

$$\min_{S_i^{log}} \|\hat{U}^T \log(\tilde{\Sigma}_i)\hat{U} - S_i^{log}\|_F$$

But since S_i^{log} must be diagonal because S_i must be diagonal, the optimal feasible solution is just the diagonal of the first term $\hat{U}^T \log(\tilde{\Sigma}_i)\hat{U}$, in other words:

$$[\hat{S}_i^{log}]_{jj} = [\hat{U}^T \log(\tilde{\Sigma}_i)\hat{U}]_{jj} \quad \text{for } j = 1 \dots h$$

This concludes the proof. In the context of our method, we note that this solution is exact when $\lambda = 0$ i.e. when regular PCA is used in the population value decomposition.

The difference of two Hermitian matrices with same eigenvectors is determined by their eigenvalues

For two Hermitian matrices A and B with common eigenvectors U, the Frobenius norm of their difference $\|A - B\|_F$ equals the ℓ^2 norm of the difference between their eigenvalues.

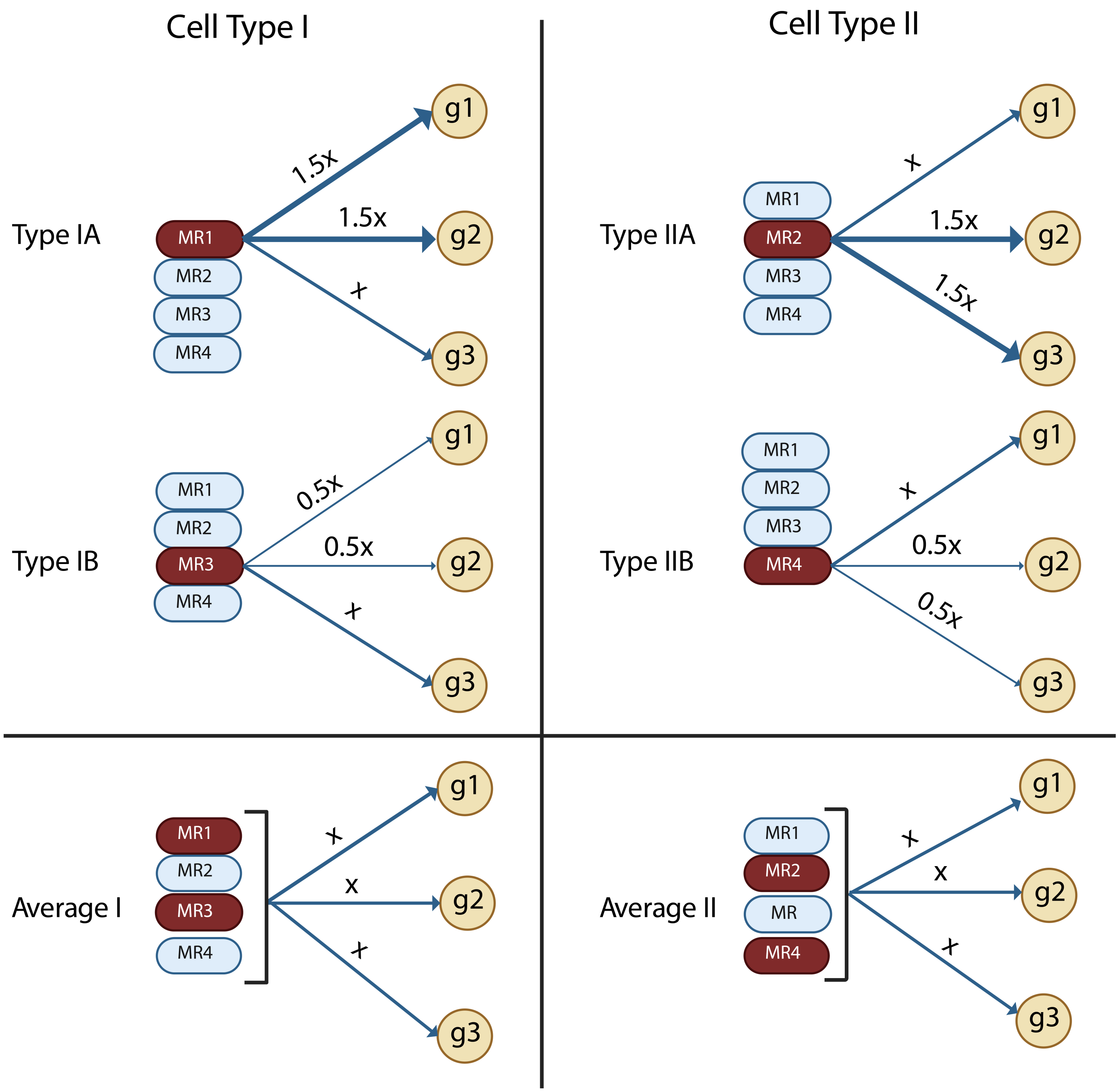

Figure 6 Gene regulatory network structure for each cell type in experiments on transcriptional data simulated via SERGIO, as seen in the top two entries of the grid. Each of the two cell types (I, II) is characterized by two cell sub-types. Each of the four cell sub-types (IA, IB, IIA, IIB) is generated by over-expression of a single master regulator gene (MR1-4) that picks out exactly two of the three downstream genes (g1, g2, g3) to be simultaneously over or under-regulated. Averaging over the cohorts corresponding to each cell sub-type, each cell type has identical expression of the downstream genes, as seen in the bottom two entries of the grid.

Proof: A and B can both be diagonalized by the shared basis U, as $A = UD_AU^*$ and $B = UD_BU^*$, where U is unitary and D_A, D_B are diagonal matrices containing the eigenvalues of A and B respectively.

An equivalent definition of the Frobenius norm is

$$\|A\|_F = (tr(A^*A))^{1/2}.$$

Now simplify $\|A - B\|_F$:

$$\|A - B\|_F = (tr((A - B)^*(A - B)))^{1/2}.$$

Substituting $A = UD_AU^*$ and $B = UD_BU^*$ gives

$$\|A - B\|_F = \left(tr\,(U^*(D_A - D_B)U)^2\right)^{1/2}.$$

Using invariance of the trace under cyclic permutations,

$$\|A - B\|_F = \left(tr(D_A - D_B)^2\right)^{1/2}.$$

Since $D_A - D_B$ is diagonal with entries $\lambda_i^{(A)} - \lambda_i^{(B)}$, this becomes

$$\|A - B\|_F = \|\lambda^{(A)} - \lambda^{(B)}\|_2.$$

Cell cohort construction

Sceodesic constructs cell cohorts by partitioning a scRNA-seq dataset into n cell cohorts $ci, i = 1, ...n$ under the premise that cells alike in terms of their transcriptional state will be grouped under the same cell cohort. In principle any single-cell clustering method can be used, but we chose Gaussian mixture models because we found this method to deliver reasonably balanced clusters, which is important because well-estimated coexpression matrices of cell cohorts are the 'base estimator' of Sceodesic. Well-balanced cell cohorts avoid extremely small clusters which would need to be heavily regularized to reside on the positive-definite manifold, distorting the spectrum of the raw covariance matrix. We describe GMMs and soft-clustering below.

To account for the noisy nature of scRNA-seq data leading to uncertainty in clustering, we took a soft-clustering approach inspired by the classic Gaussian Mixture Models (GMM). The GMM model postulates the following data-generating mechanism: cell i is drawn from a mixture of K components (latent variable z_i; scRNA-seq measurements of G genes $y_i^{(1)} \ldots y_i^{(G)}$ for cell i is given

$$z_i \sim \text{Multinoulli}(1, \ldots, K)$$

$$y_i^{(j)} \mid z_i = k \sim \text{Normal}(\mu_k^{(j)}, \Sigma_k^{(j)})$$

Here, $\mu_k = (\mu_k^{(1)}, \ldots, \mu_k^{(G)})$ is the mean expression vector for component k, and Σ_k is a $G \times G$. In our model, we constrain Σ_k to be a diagonal matrix where all mixture components have equal variance; that is, $\Sigma_k = \sigma^2 I$ for some scalar σ^2. In this case, the GMM model reduces to the classic *K-Means Clustering* model, with soft assignments.

To compute responsibilities for the soft clustering, we use the classing *K-Means* algorithm for computing cluster centers $\hat{\mu}_1, \ldots, \hat{\mu}_K$, where $\hat{\mu}_k = (\hat{\mu}_k^{(1)}, \ldots, \hat{\mu}_k^{(G)})$. The responsibility for cell i belonging in cluster k, denoted r_{ik}, is computed according to the Gaussian Mixture probability model:

$$r_{ik} = \frac{p(y_i \mid z_i = k)\, p(z_i = k)}{\sum_{t=1}^{K} p(y_i \mid z_i = t)\, p(z_i = t)}$$

$$r_{ik} = \frac{\frac{1}{\sqrt{2\pi\sigma^{2G}}} \exp\left(-(y_i - \hat{\mu}_k)^2/2\sigma^{2G}\right)\, p(z_i = k)}{\sum_{t=1}^{K} \frac{1}{\sqrt{2\pi\sigma^{2G}}} \exp\left(-(y_i - \hat{\mu}_t)^2/2\sigma^{2G}\right)\, p(z_i = t)}$$

$$r_{ik} = \frac{\exp\left(-(y_i - \hat{\mu}_k)^2/2\sigma^{2G}\right)\, p(z_i = k)}{\sum_{t=1}^{K} \exp\left(-(y_i - \hat{\mu}_t)^2/2\sigma^{2G}\right)\, p(z_i = t)}$$

To simplify computations, we treat σ^2 as a hyperparameter, denoting it as γ. We tested a range of values for γ, and found $\gamma = 0.01$ to perform reasonably well (should we show experiments for this?).

To speed up computation, we observed that cluster responsibilities were minuscule for clusters other than those corresponding to the 5 closest centroids. To speed up computation, therefore, for each cell i we defined a set of clusters C_i, consisting of the 5 clusters whose centers are closest to the observed gene expression y_i. (For example, if the 5 "nearest" clusters to cell i are $\hat{\mu}_1, \ldots, \hat{\mu}_5$, then $C_i = \{\hat{\mu}_1, \hat{\mu}_2, \hat{\mu}_3, \hat{\mu}_4, \hat{\mu}_5\}$). We then assumed responsibilities for clusters outside of C_i to be zero, and a uniform marginal distribution for the latent variable z_i within each cluster. The cluster responsibility for this simplified assignment scheme then becomes

$$r_{ik} = \frac{\exp\left(-(y_i - \hat{\mu}_k)^2/2\gamma\right) I(k \in C_i)}{\sum_{t=1}^{K} \exp\left(-(y_i - \hat{\mu}_t)^2/2\gamma\right) I(t \in C_i)}$$

where I is the indicator function. We define the responsibility matrix

$$R = \begin{pmatrix} r_{11} & r_{12} & \ldots & r_{1K} \\ r_{21} & r_{22} & \ldots & r_{2K} \\ \vdots & \vdots & \ddots & \vdots \\ r_{N1} & r_{N2} & \ldots & r_{NK} \end{pmatrix}.$$

Given the embedding matrix

$$E = \begin{pmatrix} e_{11} & e_{12} & \ldots & e_{1D} \\ e_{21} & e_{22} & \ldots & e_{2D} \\ \vdots & \vdots & \ddots & \vdots \\ e_{K1} & e_{K2} & \ldots & e_{KD} \end{pmatrix}$$

we then compute the soft embeddings assignment for the cells as a single matrix multiplication:

$$\tilde{\mathbf{E}} = \mathbf{RE}.$$

This step is motivated by the fact that GMM's probabilistic cohort assignment allows for individual cells in a cohort to differ slightly in their program weights (receive program weights from different cohorts), enhancing cell-level expressiveness and variability.

Extended Cell Type Classification Results

Below (Fig. 7), we consider the middle fifty percent of pairs of cell cohorts by Euclidean distance to show the relative outperformance of the log-Euclidean metric.

Cross-Dataset Generalization of Sceodesic Programs

We utilized Sceodesic programs fitted on the Hao et al. CITE-Seq dataset and used them as a basis for the 10x Genomics CITE-Seq dataset. On the second dataset, we clustered the data (k = 10, sample size = 10,000) and then reconstructed the cluster level covariance matrices using the log-Euclidean metric and applied the Sceodesic programs from the first dataset. We then ran the same Lasso program selection procedure with a train-test split to find the best programs for predicting surface protein expression on proteins that overlapped between the datasets.

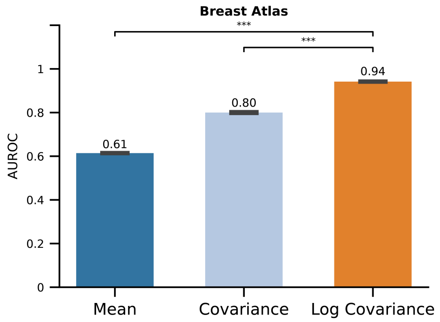

Figure 7 Extended Cell Type Classification Results: Breast Atlas Subsetted to Middle 50% of distances. Comparison of Euclidean distances between cohort means (d_μ), Euclidean distance between covariance matrices (d_Σ), and log-Euclidean distances between covariance matrices ($d_{\log \Sigma}$) as 1-D classifiers of cohort-cell type similarity as measured by AUROC for the Breast Atlas dataset, analysis restricted to middle quartiles.

Protein	R^2 Hao et al	R^2 10x Genomics
CD14	0.767	0.754
CD19	0.901	0.678
CD45RA	0.642	0.411
CD127	0.737	0.347
CD45RO	0.477	0.161
CD16	0.747	0.140
CD8A	0.434	0.109
CD15	0.115	0.099
CD25	0.232	0.036

Table 1 Cross-dataset generalization of Sceodesic programs fitted on Hao et al. to 10x Genomics Dataset. Ten randomly selected proteins.

Interpretability Analysis

We performed GSEA analysis to explicitly check the biological informativeness of Sceodesic programs. We used programs from our CITE-Seq Validation experiments. Our enrichment analysis was conducted over two popular gene set libraries (GO Biological Processes 2021, KEGG 2021 Human) as representatives of biological processes and molecular interaction networks, respectively. We considered only hits with q-values ¡ 0.05 (Benjamini-Hochberg correction). Our queries were derived from genes that were important to the programs used in downstream prediction—to be precise, we included the n largest genes that constituted less than or equal to 95% of the program mass.

**Enrichr Hit**	**Adjusted p-value**
Hematopoietic cell lineage	2.46×10^{-9}
Viral protein interaction with cytokine and cytokine receptor	4.26×10^{-7}
Cytokine–cytokine receptor interaction	3.97×10^{-6}
Complement and coagulation cascades	1.19×10^{-5}
Rheumatoid arthritis	1.91×10^{-5}
Inflammatory bowel disease	2.77×10^{-4}
Graft-versus-host disease	3.66×10^{-4}
Chemokine signaling pathway	3.66×10^{-4}
Pertussis	4.56×10^{-4}
IL-17 signaling pathway	1.31×10^{-3}

Table 2 Sceodesic Component 170 Significant Enrichr Hits

**Enrichr Hit**	**Adjusted p-value**
**Viral protein interaction with cytokine and cytokine receptor**	1.48×10^{-15}
**Cytokine–cytokine receptor interaction**	5.34×10^{-14}
**Rheumatoid arthritis**	2.46×10^{-12}
**Hematopoietic cell lineage**	4.53×10^{-12}
**Chemokine signaling pathway**	2.53×10^{-10}
**IL-17 signaling pathway**	1.88×10^{-7}
Chagas disease	3.59×10^{-7}
Primary immunodeficiency	5.57×10^{-7}
**Graft-versus-host disease**	1.03×10^{-6}
**Antigen processing and presentation**	5.06×10^{-6}

Table 3 Sceodesic Component 161 Significant Enrichr Hits

Hyperparameter Sensitivity and Runtimes

Below we provide results on the sensitivity of Sceodesic programs to varying hyperparameters. We measure this sensitivity by measuring how predictive of Sceodesic programs are under various hyperparameter settings on the protein-expression prediction task outlined in Cite-Seq Validation experiments. Because the predictability of individual proteins varies wildly, we have subsetted the robustness analysis to the ten proteins that are most predictable. We also provide the runtime under different configurations.

Below are tables showing the results of a hyperparameter sweep for the Figure 2 metric comparison. Rows in which AUROC results show 'NA' are those in which filtering of clusters for cell-type purity did not leave any clusters for analysis.

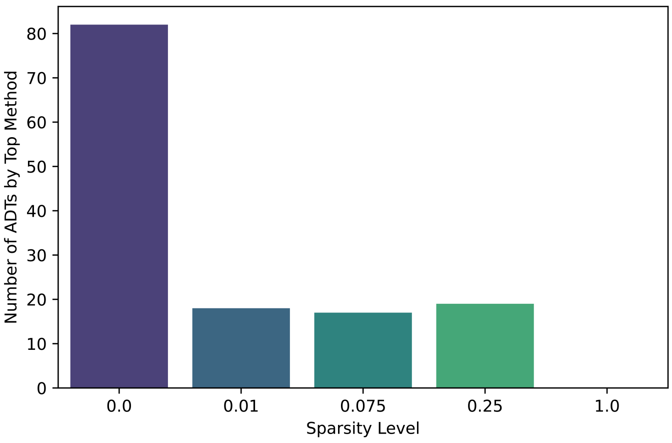

Figure 8 **Comparison of Sceodesic methods in protein-expression level prediction on CITE-Seq dataset, for varying levels of sparsity**

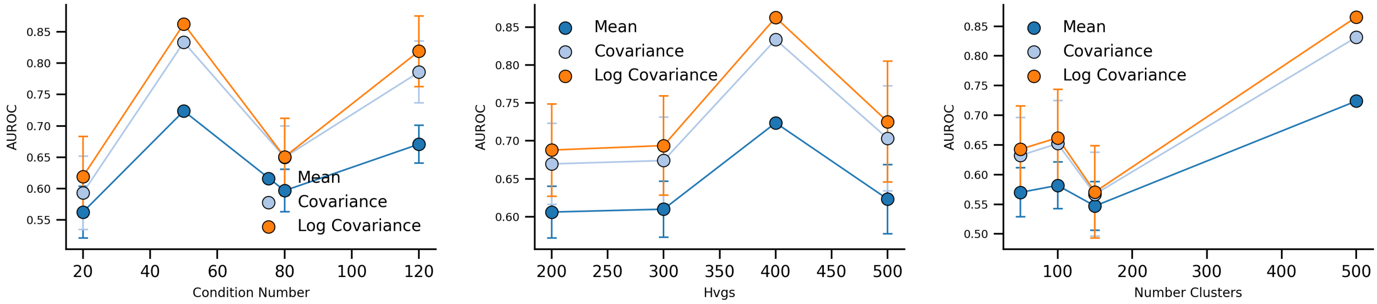

Figure 9 **Robustness of cell type classification performance in the three metrics to changing hyperparameters.**

Condition number	Data name	Hvgs	Number clusters	Auroc covariance	Auroc log covariance	Auroc mean
120	Lung Atlas	500	500	NA	NA	NA
80	Lung Atlas	300	500	0.83	0.87	0.72
80	Lung Atlas	500	500	0.83	0.87	0.72
20	Lung Atlas	500	500	0.83	0.87	0.72
20	Lung Atlas	300	500	0.83	0.87	0.72
120	Lung Atlas	300	500	0.83	0.87	0.72
120	Lung Atlas	200	500	0.83	0.87	0.72
80	Lung Atlas	200	500	0.83	0.87	0.72
120	Lung Atlas	300	150	0.49	0.48	0.49
20	Lung Atlas	500	150	NA	NA	NA
80	Lung Atlas	500	150	NA	NA	NA
120	Lung Atlas	500	150	NA	NA	NA
20	Lung Atlas	200	500	0.83	0.87	0.72
80	Lung Atlas	300	150	NA	NA	NA
20	Lung Atlas	300	150	0.50	0.50	0.52
80	Lung Atlas	200	150	0.85	0.88	0.71
120	Lung Atlas	200	150	0.49	0.48	0.49
20	Lung Atlas	500	100	NA	NA	NA
20	Lung Atlas	200	150	0.50	0.50	0.52
120	Lung Atlas	500	100	0.86	0.89	0.69
80	Lung Atlas	500	100	0.52	0.51	0.51
120	Lung Atlas	300	100	0.86	0.89	0.69
80	Lung Atlas	300	100	0.52	0.51	0.51
120	Lung Atlas	200	100	0.86	0.89	0.69
80	Lung Atlas	200	100	0.52	0.51	0.51
20	Lung Atlas	300	100	NA	NA	NA
20	Lung Atlas	200	100	0.44	0.44	0.46
80	Lung Atlas	500	50	0.54	0.49	0.52
20	Lung Atlas	300	50	0.47	0.50	0.46
120	Lung Atlas	300	50	0.88	0.93	0.73
120	Lung Atlas	200	50	0.88	0.93	0.73
80	Lung Atlas	300	50	0.54	0.49	0.52
20	Lung Atlas	500	50	0.47	0.50	0.46
80	Lung Atlas	200	50	0.54	0.49	0.52
120	Lung Atlas	500	50	0.88	0.93	0.73
20	Lung Atlas	200	50	0.47	0.50	0.46

Table 4 Robustness of cell type classification performance in Lung Atlas dataset.

Condition number	Data name	Hvgs	Number clusters	Auroc covariance	Auroc log covariance	Auroc mean
120	Heart Atlas	300	500	0.52	0.51	0.50
80	Heart Atlas	500	500	0.49	0.50	0.51
120	Heart Atlas	500	500	0.52	0.51	0.50
20	Heart Atlas	500	500	0.95	0.98	0.98
120	Heart Atlas	200	500	0.52	0.51	0.50
20	Heart Atlas	500	150	0.95	0.98	0.99
120	Heart Atlas	300	150	0.53	0.53	0.53
20	Heart Atlas	200	500	0.95	0.98	0.98
80	Heart Atlas	300	500	0.49	0.50	0.51
80	Heart Atlas	200	500	0.49	0.50	0.51
20	Heart Atlas	300	500	0.95	0.98	0.98
120	Heart Atlas	500	150	0.53	0.53	0.53
80	Heart Atlas	500	150	0.49	0.50	0.50
80	Heart Atlas	300	150	0.49	0.50	0.50
120	Heart Atlas	200	150	0.53	0.53	0.53
20	Heart Atlas	300	150	0.95	0.98	0.99
20	Heart Atlas	200	150	0.95	0.98	0.99
80	Heart Atlas	200	150	0.49	0.50	0.50
120	Heart Atlas	500	100	NA	NA	NA
120	Heart Atlas	300	100	0.96	0.98	0.99
80	Heart Atlas	300	100	0.52	0.55	0.51
80	Heart Atlas	500	100	0.52	0.55	0.51
20	Heart Atlas	300	100	0.55	0.55	0.50
20	Heart Atlas	500	100	0.55	0.55	0.50
20	Heart Atlas	200	100	0.55	0.55	0.50
120	Heart Atlas	300	50	0.48	0.49	0.47
120	Heart Atlas	500	50	0.48	0.49	0.47
80	Heart Atlas	200	100	0.52	0.55	0.51
20	Heart Atlas	300	50	0.97	0.98	1.00
80	Heart Atlas	500	50	0.50	0.51	0.50
120	Heart Atlas	200	100	0.96	0.98	0.99
20	Heart Atlas	500	50	0.97	0.98	1.00
80	Heart Atlas	300	50	0.50	0.51	0.50
80	Heart Atlas	200	50	0.50	0.51	0.50
120	Heart Atlas	200	50	0.48	0.49	0.47
20	Heart Atlas	200	50	0.97	0.98	1.00

Table 5 Robustness of cell type classification performance in Heart Atlas dataset.

Condition number	Data name	Hvgs	Number clusters	Auroc covariance	Auroc log covariance	Auroc mean
120	Embryo	500	500	NA	NA	NA
80	Embryo	500	500	NA	NA	NA
120	Embryo	200	500	0.47	0.48	0.47
80	Embryo	300	500	0.67	0.84	0.79
120	Embryo	300	500	0.47	0.48	0.47
20	Embryo	300	500	0.47	0.48	0.49
20	Embryo	500	500	0.47	0.48	0.49
80	Embryo	200	500	0.67	0.84	0.79
80	Embryo	500	150	0.49	0.50	0.49
80	Embryo	300	150	0.49	0.50	0.49
120	Embryo	300	150	0.70	0.84	0.82
120	Embryo	500	150	0.70	0.84	0.82
20	Embryo	200	500	0.47	0.48	0.49
20	Embryo	500	150	0.51	0.52	0.56
20	Embryo	300	150	0.51	0.52	0.56
120	Embryo	200	150	0.70	0.84	0.82
20	Embryo	200	150	0.51	0.52	0.56
80	Embryo	200	150	0.49	0.50	0.49
80	Embryo	500	100	NA	NA	NA
120	Embryo	500	100	0.75	0.88	0.85
120	Embryo	300	100	0.75	0.88	0.85
80	Embryo	300	100	0.49	0.49	0.49
20	Embryo	500	100	NA	NA	NA
120	Embryo	200	100	0.75	0.88	0.85
80	Embryo	200	100	0.49	0.49	0.49
20	Embryo	300	100	NA	NA	NA
120	Embryo	500	50	0.73	0.88	0.81
120	Embryo	300	50	0.73	0.88	0.81
20	Embryo	500	50	NA	NA	NA
80	Embryo	500	50	0.61	0.57	0.58
20	Embryo	200	100	0.52	0.54	0.61
80	Embryo	300	50	0.61	0.57	0.58
120	Embryo	200	50	0.73	0.88	0.81
20	Embryo	300	50	0.56	0.51	0.43
20	Embryo	200	50	0.56	0.51	0.43
80	Embryo	200	50	0.61	0.57	0.58

Table 6 Robustness of cell type classification performance in Fetal dataset.

Condition number	Data name	Hvgs	Number clusters	Auroc covariance	Auroc log covariance	Auroc mean
80	Breast Atlas	500	500	NA	NA	NA
120	Breast Atlas	500	500	NA	NA	NA
20	Breast Atlas	500	500	0.49	0.49	0.49
120	Breast Atlas	300	500	0.84	0.96	0.88
80	Breast Atlas	300	500	0.50	0.50	0.49
120	Breast Atlas	200	500	0.84	0.96	0.88
120	Breast Atlas	500	150	0.85	0.97	0.89
20	Breast Atlas	300	500	0.49	0.49	0.49
20	Breast Atlas	200	500	0.49	0.49	0.49
80	Breast Atlas	200	500	0.50	0.50	0.49
20	Breast Atlas	500	150	0.49	0.50	0.51
20	Breast Atlas	300	150	0.49	0.50	0.51
80	Breast Atlas	300	150	0.49	0.49	0.51
120	Breast Atlas	200	150	0.85	0.97	0.89
120	Breast Atlas	300	150	0.85	0.97	0.89
80	Breast Atlas	500	150	0.49	0.49	0.51
20	Breast Atlas	500	100	NA	NA	NA
80	Breast Atlas	500	100	NA	NA	NA
120	Breast Atlas	500	100	NA	NA	NA
20	Breast Atlas	200	150	0.49	0.50	0.51
80	Breast Atlas	200	150	0.49	0.49	0.51
80	Breast Atlas	200	100	0.47	0.48	0.49
80	Breast Atlas	300	100	0.47	0.48	0.49
120	Breast Atlas	200	100	0.49	0.48	0.48
20	Breast Atlas	300	100	0.87	0.98	0.91
120	Breast Atlas	300	100	0.49	0.48	0.48
20	Breast Atlas	500	50	0.94	0.99	0.93
20	Breast Atlas	200	100	0.87	0.98	0.91
120	Breast Atlas	500	50	NA	NA	NA
120	Breast Atlas	300	50	0.53	0.53	0.47
80	Breast Atlas	500	50	0.52	0.51	0.48
120	Breast Atlas	200	50	0.53	0.53	0.47
20	Breast Atlas	200	50	0.94	0.99	0.93
80	Breast Atlas	300	50	0.52	0.51	0.48
80	Breast Atlas	200	50	0.52	0.51	0.48
20	Breast Atlas	300	50	0.94	0.99	0.93

Table 7 Robustness of cell type classification performance in Breast dataset.

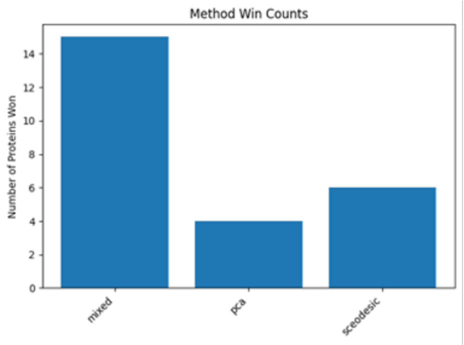

Figure 10 Mixing Sceodesic and PCA Programs: CITE-Seq analysis on 25 randomly selected proteins. Two settings are featured: First, the 10 best components from Sceodesic and the 10 best components from PCA concatenated. Second, the 20 best components from each individual method as determined by the Lasso selection procedure described in the paper. We find that using the combination leads to better performance.

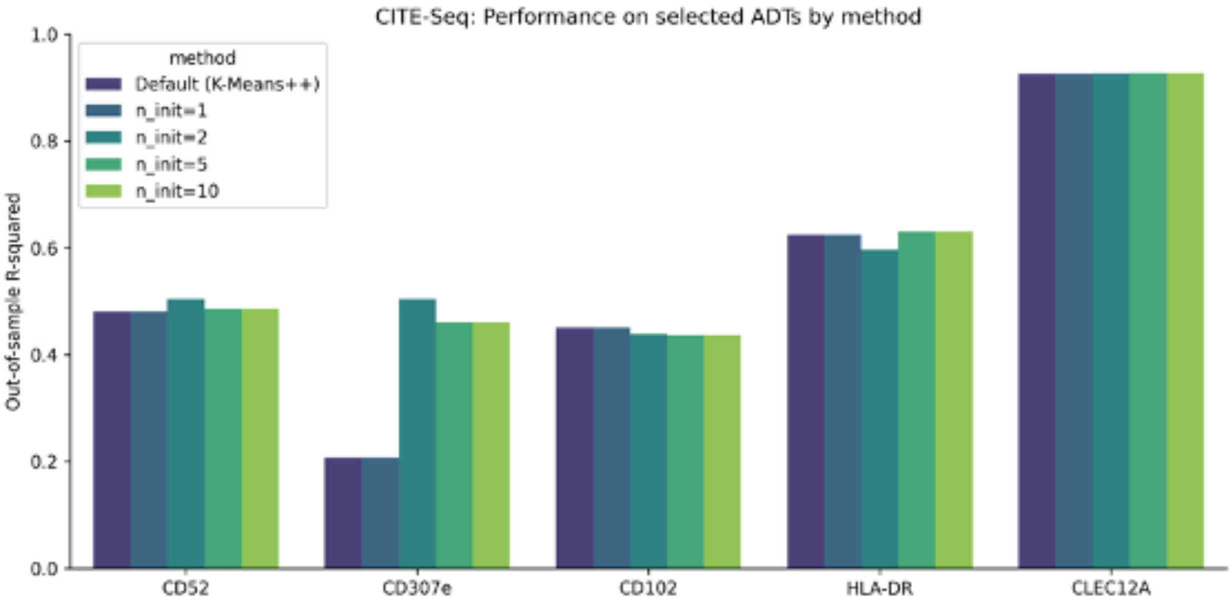

Figure 11 Simulating Cohort Misallocations: To simulate cohort misallocation, we changed the initialization of K-Means to 'random' and varied the number of initializations, reasoning that fewer initialization will lead to noisier cluster assignments. The results below show the relative performance (on the Cite-Seq validation) of Sceodesic under different clustering initializations, simulating cohort misallocation.

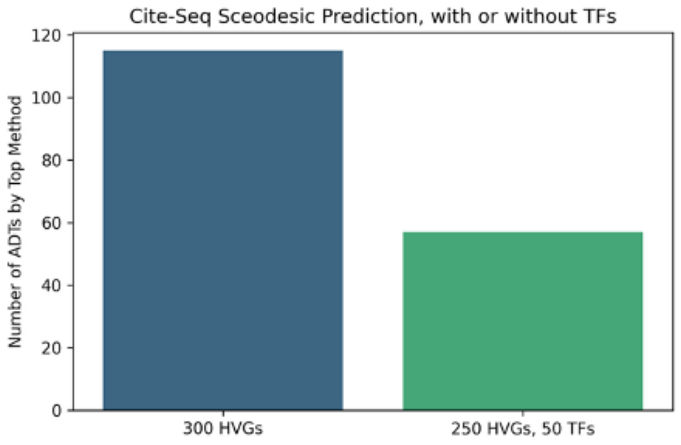

Figure 12 Protected Transcription Factor Ablation: We compared two Sceodesic runs, one with 300 HVGs and the other with 250 HVGs plus 50 most variable TFs in the dataset. We used the set of TFs available at the Riken Transcription Factor list.

Expanded Simulation Design

Expanded simulation and hyperparameter variation for SERGIO simulations. Note that groups 1 and 2 differ by covariance but not means. groups 3 and 4 differ by means. Cell states are randomly sampled unevenly and different noise parameters were simulated.

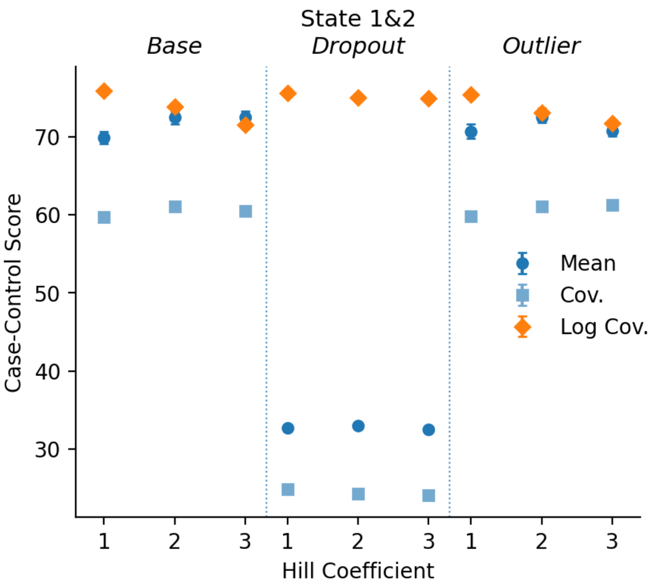

Figure 13 Base, groups 1–2.

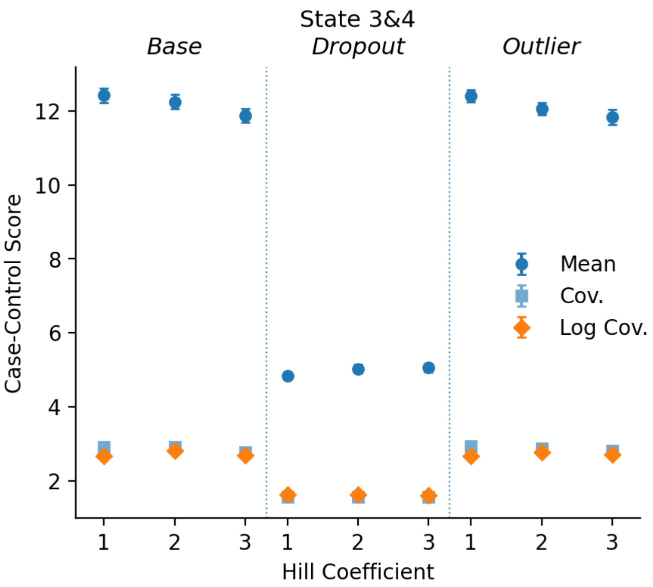

Figure 14 Base, groups 3–4.

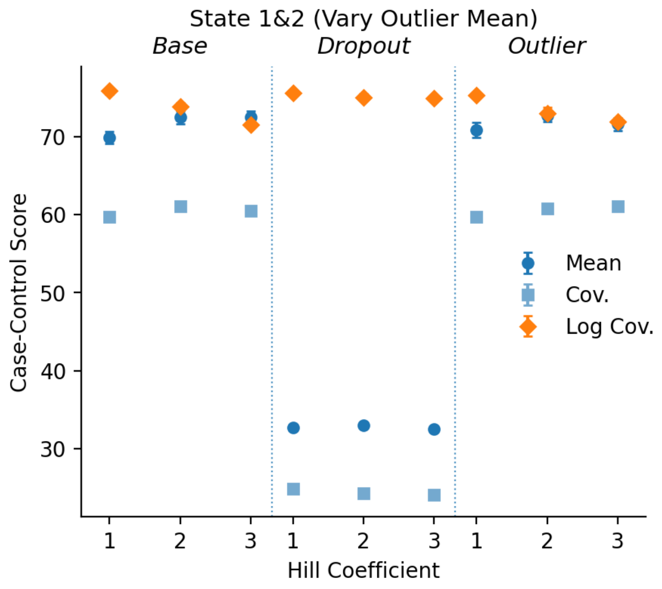

Figure 15 Vary mean, groups 1–2.

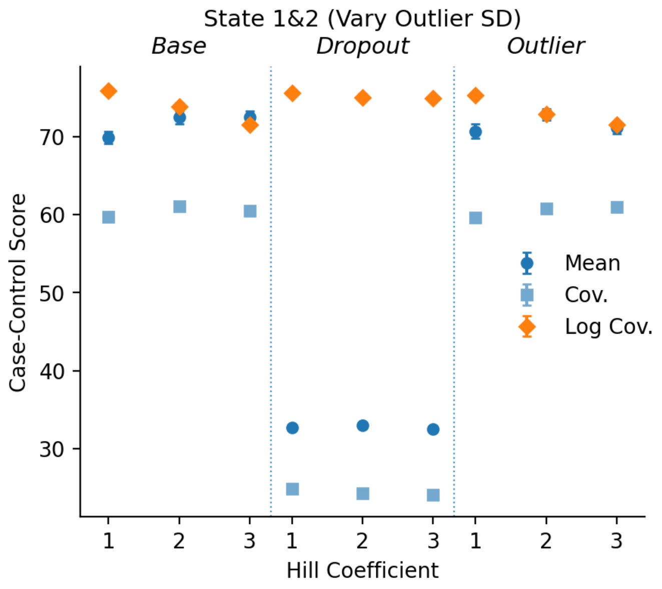

Figure 17 Vary SD, groups 1–2.

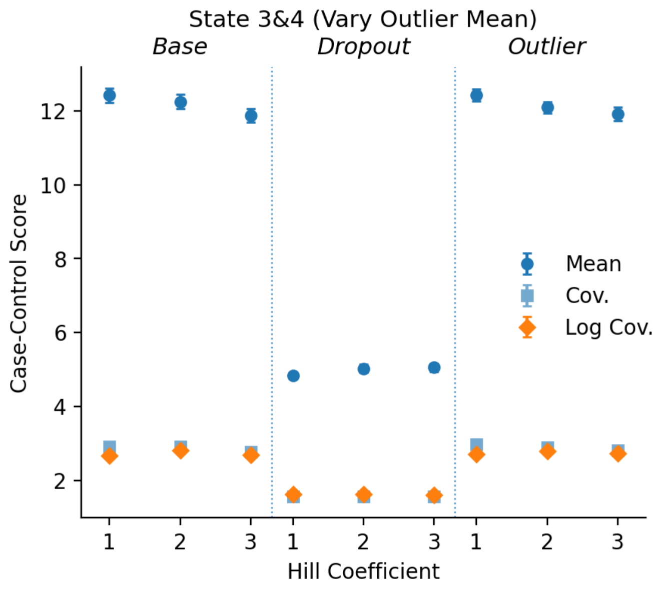

Figure 16 Vary mean, groups 3–4.

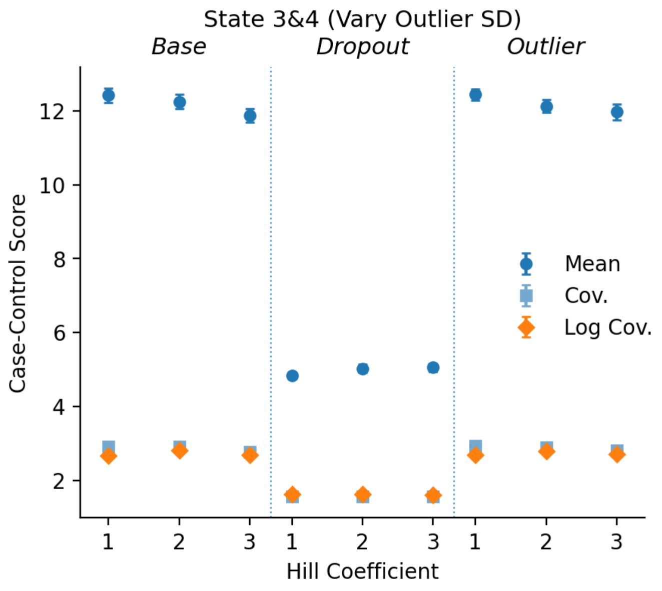

Figure 18 Vary SD, groups 3–4.

Sceodesic Parameter Sensitivity

In tables A.8 and A.9, we selected the middle 25% of proteins by predictability (according to the base configuration of Sceodesic) as indicative and computed R-squared averaged over proteins, varying hyperparameters.

Cohorts	R^2-Test
30	0.25
50	0.24
100	0.26
200	0.28

Table 8 Cohort size sweep.

Sparsity	R^2-Test
0.00	0.27
0.01	0.26
0.07	0.27
0.25	0.24
1.00	0.08

Table 9 Sparsity sweep.

Table 10 Sceodesic runtime under various hyperparameter configurations.

HVGs	Cohorts	Sparsity	Condition number	Soft	N-init	Protected-TF	Runtime (s)
200	100	0.00	50	True	10	False	423.22
300	30	0.00	50	True	10	False	274.12
300	50	0.00	50	True	10	False	320.39
300	100	0.00	20	True	10	False	533.03
300	100	0.00	50	True	1	False	234.20
300	100	0.00	50	True	2	False	254.94
300	100	0.00	50	True	5	False	308.91
300	100	0.00	50	True	10	False	477.39
300	100	0.00	50	True	10	True	245.16
300	100	0.00	80	True	10	False	374.35
300	100	0.00	120	True	10	False	360.89
300	100	0.00	250	True	10	False	240.81
300	100	0.00	500	True	10	False	264.66
300	100	0.00	1000	True	10	False	353.31
300	100	0.00	10000	True	10	False	420.79
300	100	0.01	50	True	10	False	308.46
300	100	0.07	50	True	10	False	380.88
300	100	0.25	50	True	10	False	367.09
300	100	1.00	50	True	10	False	551.52
300	200	0.00	50	True	10	False	732.08
400	100	0.00	50	True	10	False	270.30
500	100	0.00	50	True	10	False	381.10

Ablations

1. Condition number normalization removal: We compared the typical range of condition numbers for Sceodesic (around the default value of 50) to a condition number setting of 10000, which is almost equivalent to not having any spectral constraint. The latter performed the worst of all choices. 2. Log-Euclidean reconstruction/log-map ablation. We used the Frobenius norm for reconstruction instead of the log-Euclidean map. 3. Local to global aggregation ablation. Instead of learning cross-cohort programs, we concatenated an equal number of the highest variance eigenvectors from each cohort to form a global basis.

We then reran the Cite-Seq protein level prediction task, in which we measured how well the top ten programs generated by a given method are at facilitating the prediction of protein expression levels in a linear regression setting.

Regulon Recovery

Fetal Time Course Experiments

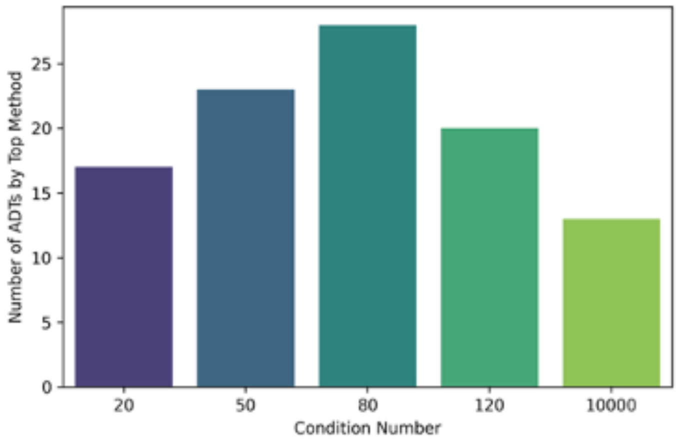

Figure 19 Without Condition number normalization.

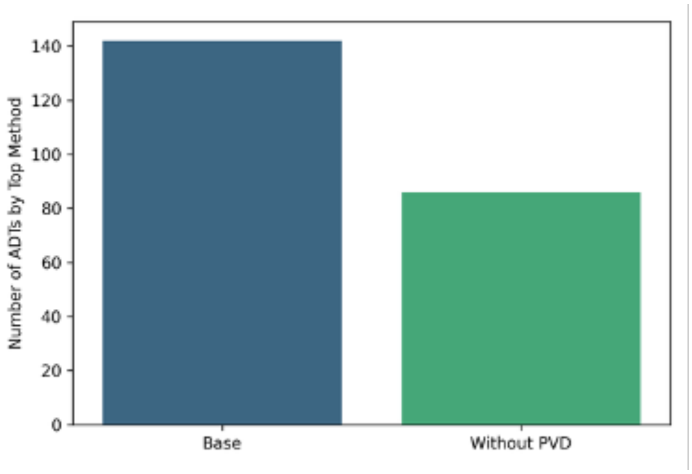

Figure 20 Without PVD

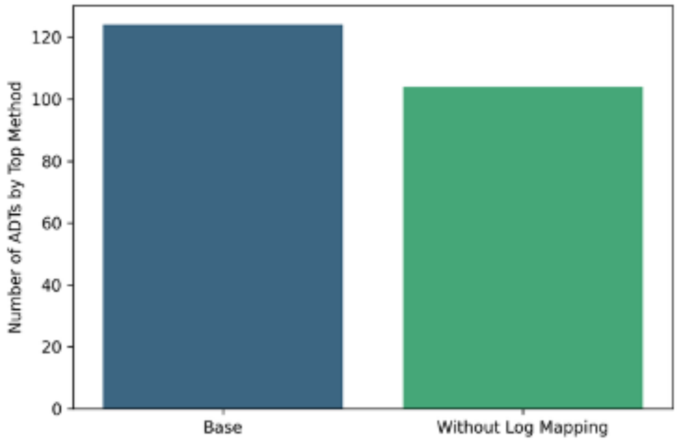

Figure 21 Without Log Mapping

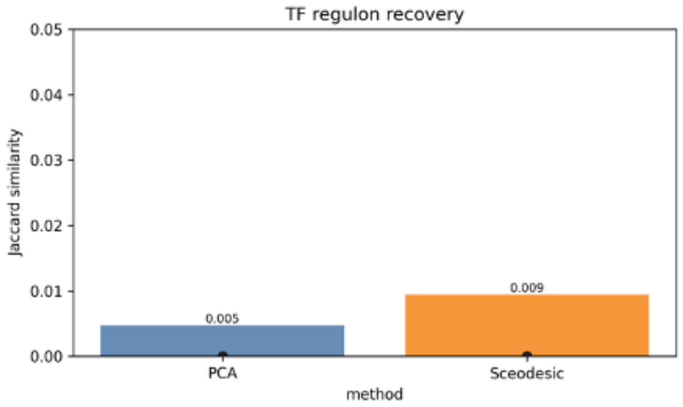

Figure 22 Selecting programs where a transcription factor was in the top 25 genes by program weight we assessed if the remaining genes in the top 25 set were listed as part of TF-regulon pairs for that transcription factor. The metric we computed for the program was the Jaccard similarity between the regulons from the program and the full set of regulons from TRRUST for that TF

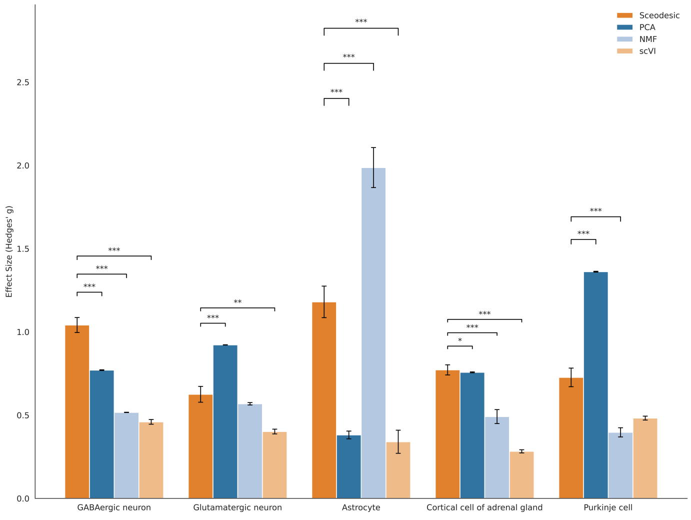

Figure 23 Absolute value of Hedges' g effect size for change in program expression level from earliest time point to latest time point available. Computed for the program with maximum Spearman correlation with time course.
